# Homeostatic regulation through GABA and acetylcholine muscarinic receptors of motor trigeminal neurons following sleep deprivation

**DOI:** 10.1007/s00429-017-1392-4

**Published:** 2017-03-15

**Authors:** Hanieh Toossi, Esther Del Cid-Pellitero, Barbara E. Jones

**Affiliations:** Department of Neurology and Neurosurgery, McGill University, Montreal Neurological Institute, 3801 University Street, Montreal, QC H3A 2B4 Canada

**Keywords:** GABA_A_, GABA_B_, AChM2, Mice, Muscle atonia, Waking

## Abstract

Muscle tone is regulated across sleep-wake states, being maximal in waking, reduced in slow wave sleep (SWS) and absent in paradoxical or REM sleep (PS or REMS). Such changes in tone have been recorded in the masseter muscles and shown to correspond to changes in activity and polarization of the trigeminal motor 5 (Mo5) neurons. The muscle hypotonia and atonia during sleep depend in part on GABA acting upon both GABA_A_ and GABA_B_ receptors (Rs) and acetylcholine (ACh) acting upon muscarinic 2 (AChM2) Rs. Here, we examined whether Mo5 neurons undergo homeostatic regulation through changes in these inhibitory receptors following prolonged activity with enforced waking. By immunofluorescence, we assessed that the proportion of Mo5 neurons positively stained for GABA_A_Rs was significantly higher after sleep deprivation (SD, ~65%) than sleep control (SC, ~32%) and that the luminance of the GABA_A_R fluorescence was significantly higher after SD than SC and sleep recovery (SR). Although, all Mo5 neurons were positively stained for GABA_B_Rs and AChM2Rs (100%) in all groups, the luminance of these receptors was significantly higher following SD as compared to SC and SR. We conclude that the density of GABA_A_, GABA_B_ and AChM2 receptors increases on Mo5 neurons during SD. The increase in these receptors would be associated with increased inhibition in the presence of GABA and ACh and thus a homeostatic down-scaling in the excitability of the Mo5 neurons after prolonged waking and resulting increased susceptibility to muscle hypotonia or atonia along with sleep.

## Introduction

Motor activity and muscle tone are regulated across the sleep-waking cycle. Muscle tone is maximal in alert waking, reduced in slow wave sleep (SWS) and absent in paradoxical or REM sleep (PS or REMS) (Jouvet 1967; Chase 1983). During this cycle, motor neurons in the brainstem and spinal cord fire during waking, decrease their firing during SWS and cease firing during REMS. Intracellular recordings showed that the motor trigeminal, 5th nerve (Mo5) neurons are slightly hyperpolarized during SWS and strongly hyperpolarized during REMS relative to active waking (Chandler et al. 1980; Chase et al. 1980), as are also motor neurons of the motor hypoglossal, 12th nerve (Mo12) and spinal cord (Morales and Chase 1978; Fung and Chase 2015). The post-synaptic inhibition of the Mo5 and motor spinal neurons was shown to be blocked by strychnine and thus dependent upon the inhibitory neurotransmitter, glycine and its postsynaptic receptors (Soja et al. 1987; Chase et al. 1989). Yet, studies also indicated that the atonia of the masseter muscles could not be fully blocked by antagonism of glycine receptors with strychnine alone but only by the additional antagonism of GABA receptors, and not solely of GABA_A_Rs with bicuculline but only additionally of GABA_B_Rs with baclofen (Soja et al. 1987; Brooks and Peever 2008, 2012). It was also found that glycine and GABA_A_ receptor antagonism increased tone during waking and SWS as well as REMS in both masseter and genioglossus muscles (Soja et al. 1987; Brooks and Peever 2008; Morrison et al. 2003). In the genioglossus muscles, it was discovered that the muscle atonia during REMS could be selectively prevented by antagonism of acetylcholine (ACh) muscarinic (M) receptors using scopolamine or by blocking the inhibitory pathway through the G-protein coupled inwardly rectifying potassium channel (GIRK) to which the AChM2R is linked (Grace et al. 2013a, b). Given the demonstrated post-synaptic inhibitory action of the AChM2R through GIRK channels in motor neurons (Chevallier et al. 2006; Miles et al. 2007; Zhu et al. 2016), the demonstrated presence of AChM2Rs on Mo5 neurons (Hellstrom et al. 2003; Brischoux et al. 2008) and the established role of ACh in PS or REMS (Jones 1991), it would appear that such AChM2R-mediated inhibition could well occur during REMS. These results indicate that motor neurons in the brainstem, including the Mo5 neurons, are inhibited during sleep by both ionotropic glycine/GABA receptors and metabotropic GABA_B_ and AChM2 receptors.

The sleep-waking cycle is known to be regulated in a homeostatic manner, since sleep deprivation (SD) with enforced waking leads to a subsequent decrease in waking and increase in sleep, including both SWS and REMS (Borbely et al. 1984). The homeostatic drive is evident following SD in both cortical activity, as an increase in slow wave activity, and peripheral muscle tone, as an increase in muscle atonia (Werth et al. 2002; Borbely and Achermann 1999; Tobler and Borbely 1986), which suggest homeostatic changes in central neurons, including motor neurons.

The activity of individual neurons is also known to be regulated in a homeostatic manner, such that prolonged increases in activity can lead to decreases in activity associated with decreases in excitability of the individual neurons (Turrigiano 1999). These cell autonomous adjustments have been referred to as homeostatic synaptic scaling that is triggered and sensed by altered levels of neuronal discharge and/or membrane polarization and is mediated by global changes in excitatory and/or inhibitory receptors in individual neurons (Turrigiano et al. 1998; Kilman et al. 2002; Marty et al. 2004). In both in vitro and in vivo studies, prolonged activity has been shown to lead to increases in GABA_A_ receptors that are associated with increases in post-synaptic inhibitory currents (Nusser et al. 1998; Marty et al. 2004). We thus hypothesized that prolonged activity of motor neurons during enforced waking with SD could lead to homeostatic increases in inhibitory receptors, including GABA_A_, GABA_B_ and AChM2 receptors. We investigated this possibility by quantitative assessment of the receptors in immunofluorescent-stained sections from brains of mice following enforced waking with SD compared to those following normal or enhanced sleep with sleep control (SC) and sleep recovery (SR) conditions. The work has now been published in abstract form (Toossi et al. 2016a).

## Materials and methods

All procedures were performed according to the guidelines of the Canadian Council on Animal Care and approved by the animal care committee of McGill University.

### Sleep deprivation and recovery experimental procedures

A total number of 12 adult male mice (C57BL/6, 20-25g) were received from the supplier (Charles River) and housed individually under 12-h light : 12-h dark schedule (lights on from 7:00 to 19:00) at 22 °C ambient temperature and with unlimited access to food and water at all time. Animals were maintained in their home cages for the duration of the experiment and recorded by video and telemetric electroencephalogram (EEG) using HomeCageScan software (HomeCageScan™ 3.0; Clever Systems) (del Cid-Pellitero, Plavski and Jones, unpublished results). For telemetric recording of the EEG, two electrodes were placed symmetrically over parietal cortex along with two for reference over cerebellum and were connected by wires to a transmitter (F20-EET, Data Sciences International, DSI) implanted subcutaneously along the flank. Following surgery, the mice were allowed 1 week to recover.

The three experimental groups were composed of: (1) sleep control (SC) mice allowed to sleep undisturbed for 2 h from ~14:00 to ~16:00 (~ZT 7–9) (*n* = 3), (2) sleep deprived mice (SD) maintained awake for 2 h (*n* = 3) or 4 h (*n* = 3) from ~12:00 to ~16:00 (~ZT 5–9) and (3) sleep recovery (SR) mice allowed to sleep for 2 h from ~14:00 to ~16:00 (~ZT 7–9) after being maintained awake for 4 h prior to euthanasia (*n* = 3). The mice were maintained awake by gentle stimulation with a soft paintbrush of the whiskers each time the mouse appeared to be preparing to sleep. Mice were immediately anaesthetized after the experimental period at ~16:00 (~ZT 9) with sodium pentobarbital (Euthanyl, 100 mg ⁄ kg; Bimeda-MTC) and perfused transcardially with 30 ml of cold saline followed by 200 ml of 3% paraformaldehyde solution. Brains were removed, post-fixed in 3% paraformaldehyde for 1 h at 4°C, then placed in 30% sucrose solution at 4 °C for 2 days, frozen to −50 °C and stored at −80 °C.

Sleep and waking were scored by behavior and EEG using HomeCageScan software.

### Immunohistochemistry

Brains were cut and processed for fluorescent staining in batches of 2–4 that included mice from SC, SD and/or SR groups of the same experimental session or period. Coronal sections were cut through the brainstem on a freezing microtome at a 20 µm thickness and collected in 5 adjacent series, such that sections were separated by 100 µm intervals in each series. Free floating sections were rinsed in 0.1 M trizma saline buffer (pH 7.4), then incubated in 6% normal donkey serum buffer for 30 min and subsequently incubated overnight at room temperature in a buffer containing 1% normal donkey serum with one of the primary antibodies. The following antibodies were employed: mouse anti-GABA_A_R β2-3-chain (clone BD17, 1:100, Millipore (Chemicon), CAT# MAB 341, RRID: AB_2109419), guinea pig anti-GABA_B_R1 (1:2500, Millipore (Chemicon), CAT# AB1531, RRID: AB_2314472) or rabbit anti-AChM2R (1:600, Sigma, CAT# M9558, RRID: AB_260727). Subsequently, sections were incubated at room temperature for 2 h in Cyanine-conjugated (Cy3) secondary antibodies from donkey (Jackson ImmunoResearch Laboratories): Cy3-conjugated anti-mouse (1:1000, CAT# 715-165-150, RRID: AB_2340813), Cy3-conjugated anti-guinea pig (1:1000, CAT# 706-165-148, RRID: AB_2340460) or Cy3-conjugated anti-rabbit (1:1000, CAT# 711-165-152, RRID: AB_2307443). Sections were subsequently stained with green fluorescent Nissl stain (FNS) (1:2000, CAT # N-21,480, Molecular Probes) for 20 min. Finally, sections were rinsed, mounted and coverslipped with glycerol (Fisher).

All the receptor antibodies employed were produced and characterized years ago and have since been in use over many years (as cited herewith). For the GABA_A_R, the antibody against the β2-3-chain was employed because it stains the most prevalent types of GABA_A_ heterodimeric receptors on neurons in the brain and stains clusters of the GABA_A_R on the plasma membrane, which are associated with functional inhibitory post-synaptic currents (IPSCs) (Fritschy and Mohler 1995; Wan et al. 1997; Nusser et al. 1998). For the GABA_B_R, the antibody against the R1 subunit was employed because it also stains vast populations of neurons in the brain and is visible over cytoplasmic organelles and the plasma membrane, where it forms together with the R2 subunit the heteromeric functional receptor (Margeta-Mitrovic et al. 1999; Filippov et al. 2000; Straessle et al. 2003). For the AChM2R, the antibody employed was shown to be highly specific and to stain most prominently the surface membranes of all cholinergic as well as diverse noncholinergic neurons in the brain (Levey et al. 1991).

### Immunohistochemical image analysis

Stained sections were viewed using a Leica DMLB microscope equipped with x/y/z motorized stage, a digital camera (Orca-R^2^, C10600-10B, Hamamatsu photonics K.K) and fluorescence filters for excitation and emission of Cy2 and Cy3 dyes. Images were acquired and analyzed using the Optical Fractionator Probe of StereoInvestigator (MicroBrightField, MBF), which allows unbiased, systematic random sampling of a region of interest for cell number estimation or measurement of specific parameters, including luminance. Given application of systematic random sampling for examining and marking cells at high magnification and the subsequent measurement of fluorescence intensity of the receptor staining in the marked receptor + cells employed here, double blind procedures were not applied in this process. In each series, three sections (at 100 µm intervals) were taken through the Mo5 nucleus. In each section, a contour was traced around the Mo5 nucleus under a 5× objective. Multi-channel image stacks with 0.5 μm thickness of optical sections were then acquired under a 40× objective through the mounted histological section of approximately 15 µm thickness. For the image acquisition, the exposure time and contrast were set for each receptor series according to the parameters which provided suitable images of the brightest to the dimmest fluorescence. These parameters were maintained for all sections and mice across all groups for each receptor series. In the Optical Fractionator Probe, a grid size of 200 × 200 μm^2^ and a counting frame of 120 × 120 μm^2^ were used in the image acquisition and assessment with marking of positively labeled cells. Across the three sections, approximately 21 counting frames for Mo5 neurons were acquired and analyzed per series. Within these images, cell somata with visible nuclei located > 1 µm below the surface of the section were marked for counting, thus through 14 µm of the section. The motor neurons were identified in the FNS stained sections by their distinct morphology and thus referred to as MoFNS-positive (+). The average number of MoFNS + cells counted across series on one side was 32.86 ± 1.16 (mean ± SEM). For marking of cells positively stained for receptors, the immunostaining for the GABA_A_, GABA_B_ and AChM2 receptors on the membrane or over the cytoplasm of the MoFNS + somata was assessed by moving through the z stack of 0.5 μm thick optical sections of each MoFNS + cell soma. Estimated total numbers of double-labeled cells were computed for each series (GABA_A_R-FNS, GABA_B_R-FNS, and AChM2R-FNS) and expressed as % of MoFNS + cell population per series through the Mo5 nucleus.

Luminance measurements of the receptor immunofluorescence were performed in the marked double-labeled cells, comprising 5–10 cells per animal for GABA_A_R+ and 10 cells per animal for GABA_B_R+ and AChM2R+. The images had been acquired with the 8-bit setting of the digital camera, which thus provides converted gray scale images of the fluorescence with arbitrary units of 0–256 for luminance measures. Image acquisition was made as rapidly as possible for each cell so as to avoid bleaching of the fluorescence. As it was previously described in (Toossi et al. 2016b), two approaches were used to measure the intensity of receptor immunofluorescence over the membrane vs. that over the cytoplasm plus membrane of the soma. A rectangular box sized at 1.5 × 0.3 μm^2^ was placed over the plasma membrane for luminance measurement of the membrane fluorescence and another box over the nucleus for luminance measurement and subsequent subtraction of the background fluorescence in each cell. A donut-shaped contour was drawn around the perikaryon to include the cytoplasm and plasma membrane for luminance measurement of the cytoplasm plus membrane and another spherical contour drawn around the nucleus for measurement and subtraction of background fluorescence in each cell.

Cell counts and luminance measurements were analyzed across experimental groups for each receptor series (GABA_A_, GABA_B_ or AChM2R) using one-way analysis of variance (ANOVA) followed by post-hoc paired comparisons with Fisher’s LSD using SYSTAT (SYSTAT Software Inc., version13). In an initial analysis, the proportion of GABA_A_R+/MoFNS + neurons was found to differ significantly across the original 4 groups (F_(3,8)_ = 4.27, *p* = 0.045), however to not differ significantly between the SD2 and SD4 groups (*post-hoc* paired comparisons, *p* = 0.387), for which reason they were subsequently combined into one SD group for subsequent analysis and presentation of results. In addition, the proportion of GABA_B_R+ and AChM2R + of the MoFNS + neurons was 100% in all groups.

For higher resolution, images were also acquired using an LSM 710 confocal laser scanning microscope equipped with Ar 488-nm and He–Ne 543-nm lasers for excitation and emission of Cy2 and Cy3 dyes. Images were acquired under 63× oil objectives with a 1.0 airy unit pinhole size for each channel and 0.5 μm thick optical section.

Image plates were prepared and composed using Adobe Creative Suite (CS4, Adobe System) from fluorescent microscopic images which were used for quantitative measurements and from confocal images which were used for qualitative assessment with higher resolution of the receptor immunostaining. In all cases as stated above, the parameters of acquisition including exposure time and contrast, were set at the beginning for each receptor series and maintained the same across sections, mice and groups. In no case were brightness or contrast adjusted on individual images. However, for the fluorescent microscopic images of the GABA_A_R immunofluorescence, which was quite dim in the SC brains, the brightness and contrast were enhanced uniformly across the three images of the three mice for better visibility of the receptor immunostaining in the image plate.

## Results

### Sleep-wake states across groups

**Fig. 1:**
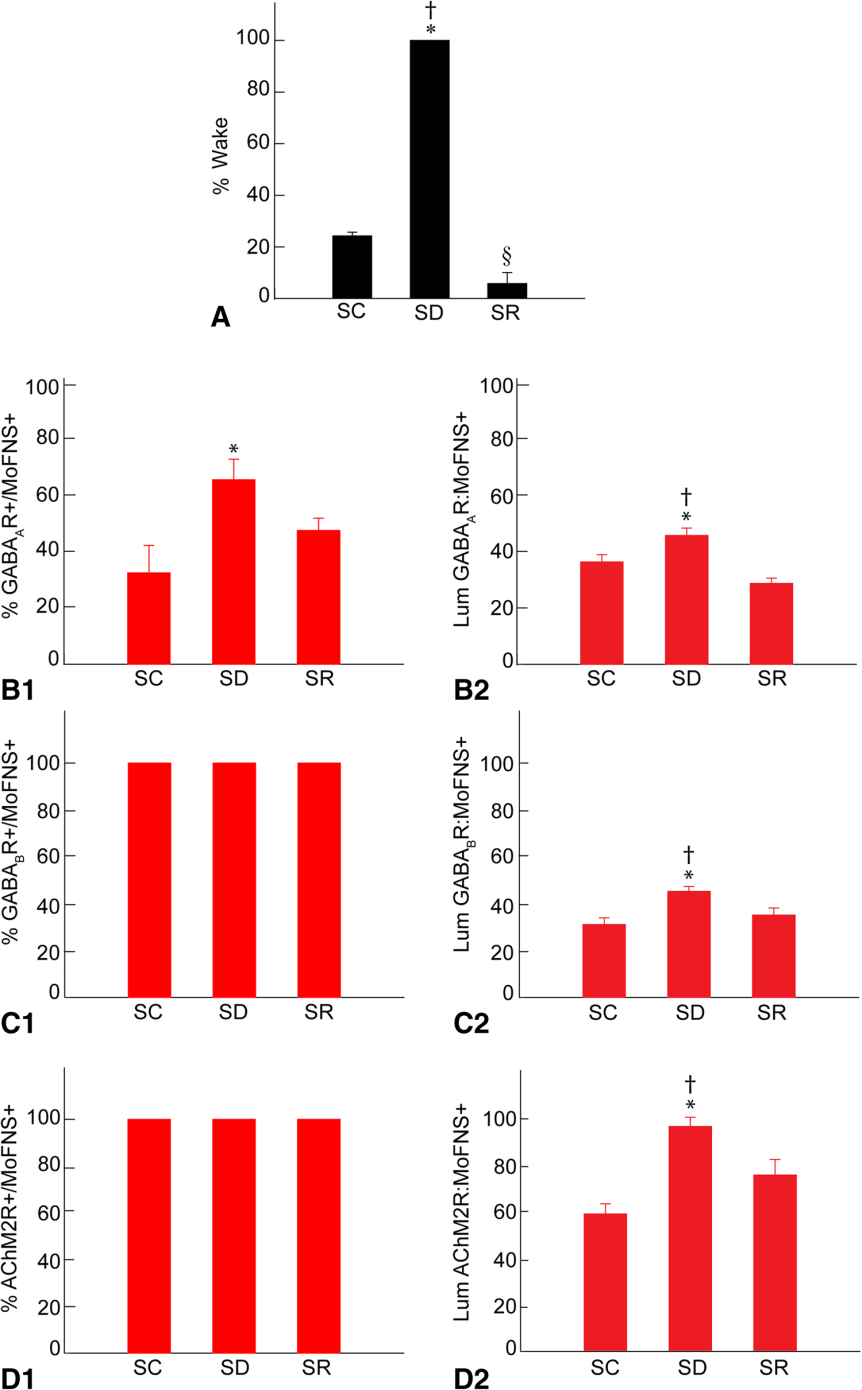
Sleep-wake states and GABA and AChM2 receptors in Mo5 neurons across groups. **A** The percentage of time spent in wake during the 2 h preceding termination differed significantly across groups, being higher in SD as compared to SC and SR and lower in SR as compared to SC. **B** The % of MoFNS + neurons which were positively immunostained for the GABA_A_R (+) differed significantly between groups, being greater in SD as compared to SC (**B1**). The luminance of the GABA_A_R immunofluorescence on GABA_A_R+/MoFNS + neurons differed significantly, being higher in SD as compared to SC and SR (**B2**). **C** All Mo5 neurons were positively immunostained for the GABA_B_R in all groups (**C1**). The luminance of the GABA_B_R in the MoFNS + neurons differed significantly and was higher in SD as compared to SC and SR (**C2**). **D** All the Mo5 neurons were positively immunostained for the AChM2R in all groups (**D1**). The luminance of the AChM2R on MoFNS + neurons differed significantly, being higher following SD as compared to SC and SR (**D2**). Note that the changes in GABARs and AChM2Rs on Mo5 neurons parallel the % Wake across groups. *Bars* represent Mean ± SEM for each group, *indicates significant difference of SD relative to SC, ^†^indicates significant difference of SD relative to SR, ^§^indicates significant difference of SR relative to SC (*p* < 0.05), according to *post hoc* paired comparisons following one-way ANOVA

Whereas mice are normally awake a small percentage of the time during the day, as evident in the SC (*n* = 3) group, the mice in the SD group (*n* = 6) were maintained awake ~ 100% of the time according to behavioral and EEG criteria during the 2 h prior to termination at 16:00 (F_(2 ,9)_ = 1381, *p* < 0.001; *n* = 12) (Fig. 1A). During this time, the SD mice engaged in walking, rearing, eating and grooming behaviors, which were all significantly increased relative to the SC mice, or remained still while awake with eyes open. Mice in the SC group were awake ~ 24% of the time (23.79 ± 1.02%, Mean ± SEM) and mice in the SR group ~ 6% of the time (5.67 ± 2.63%, *n* = 3), which was significantly less than in the SC group (*post hoc* paired comparison, *p* < 0.001). Being undisturbed, the mice in the SC group, thus slept ~ 76% of the time and mice in the SR group, which were allowed 2 h recovery sleep after 4 h SD, slept ~ 94% of the time, indicating a homeostatic response to SD. The major proportion of time for the SC and SR groups was spent in NREMS (66.93 ± 1.71, and 82.29 ± 4.07%, respectively), and a minor proportion in REMS (9.28 ± 0.89 and 12.03 ± 0.87%, respectively). Both NREM and REMS were significantly increased during SR relative to SC.

### GABA_A_Rs on Mo5 neurons after SD and SR

**Fig. 2:**
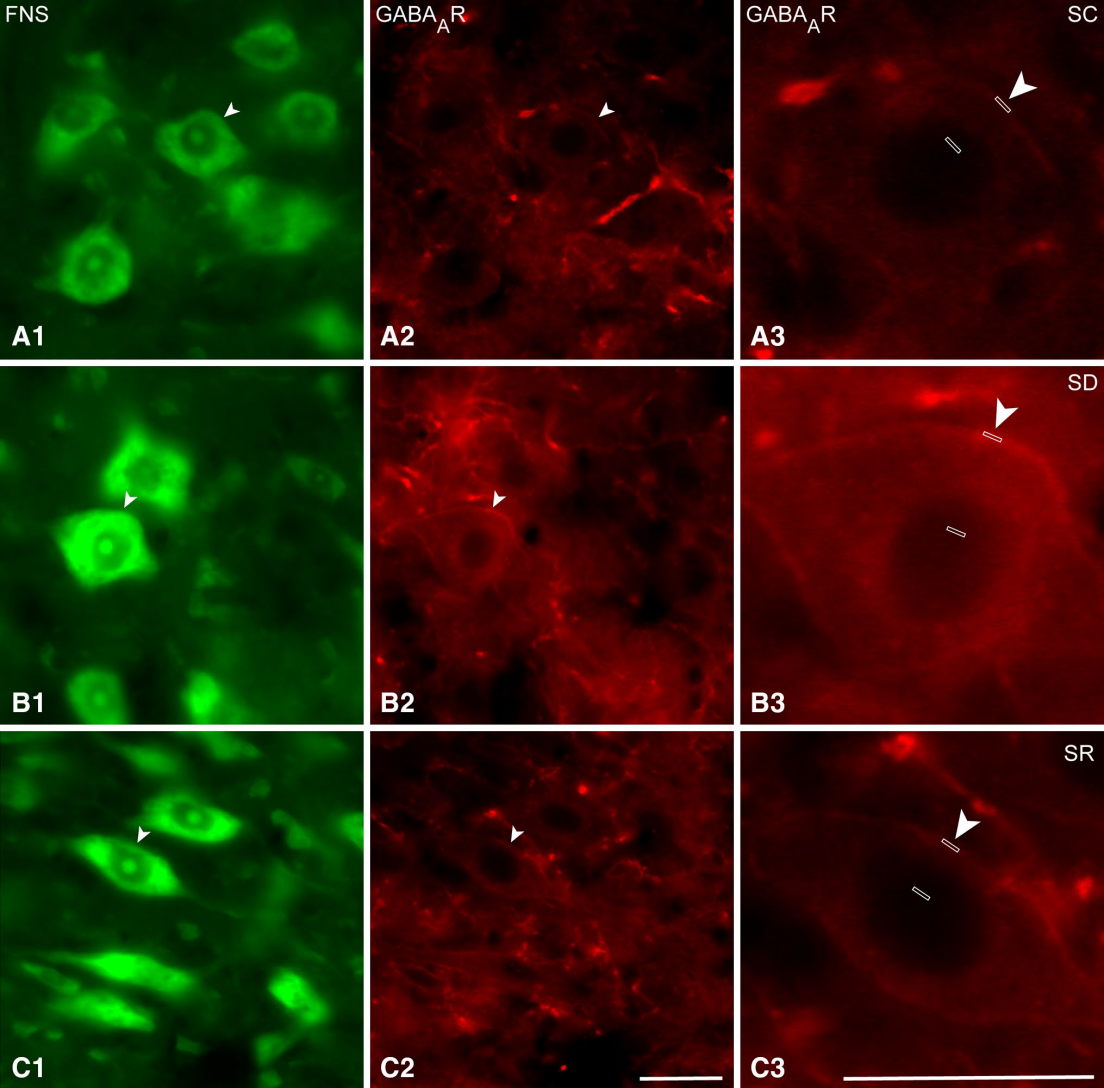
Fluorescent microscopic images of GABA_A_Rs in Mo5 neurons across groups. Images of single optical sections show several large motor neurons stained for Nissl with FNS (*green*, **A1, B1, C1**) and immunostained for the GABA_A_R (*red*, **A2, B2, C2**) within the Mo5 nucleus of each mouse. Through the one optical section shown, GABA_A_R immunofluorescence is visible over the plasma membrane of one Mo5 neuron (*arrowhead*) which is magnified on the *right* (~3×, **A3, B3, C3**) for each mouse. In the SC mouse (MST23), the GABA_A_R immunofluorescence is very dim, whereas in the SD mouse (MST20), it is very bright and in the SR mouse (MST22), less bright than in the SD mouse. For measurement of the GABA_A_R fluorescence intensity, a small *rectangular box* was placed over the plasma membrane and for that of the background fluorescence of the same cell, another over the nucleus to be subtracted from that of the plasma membrane (**A3, B3, C3**, see Methods). *Scale bars* 20 μm. Optical image thickness: 0.5 μm

**Fig. 3:**
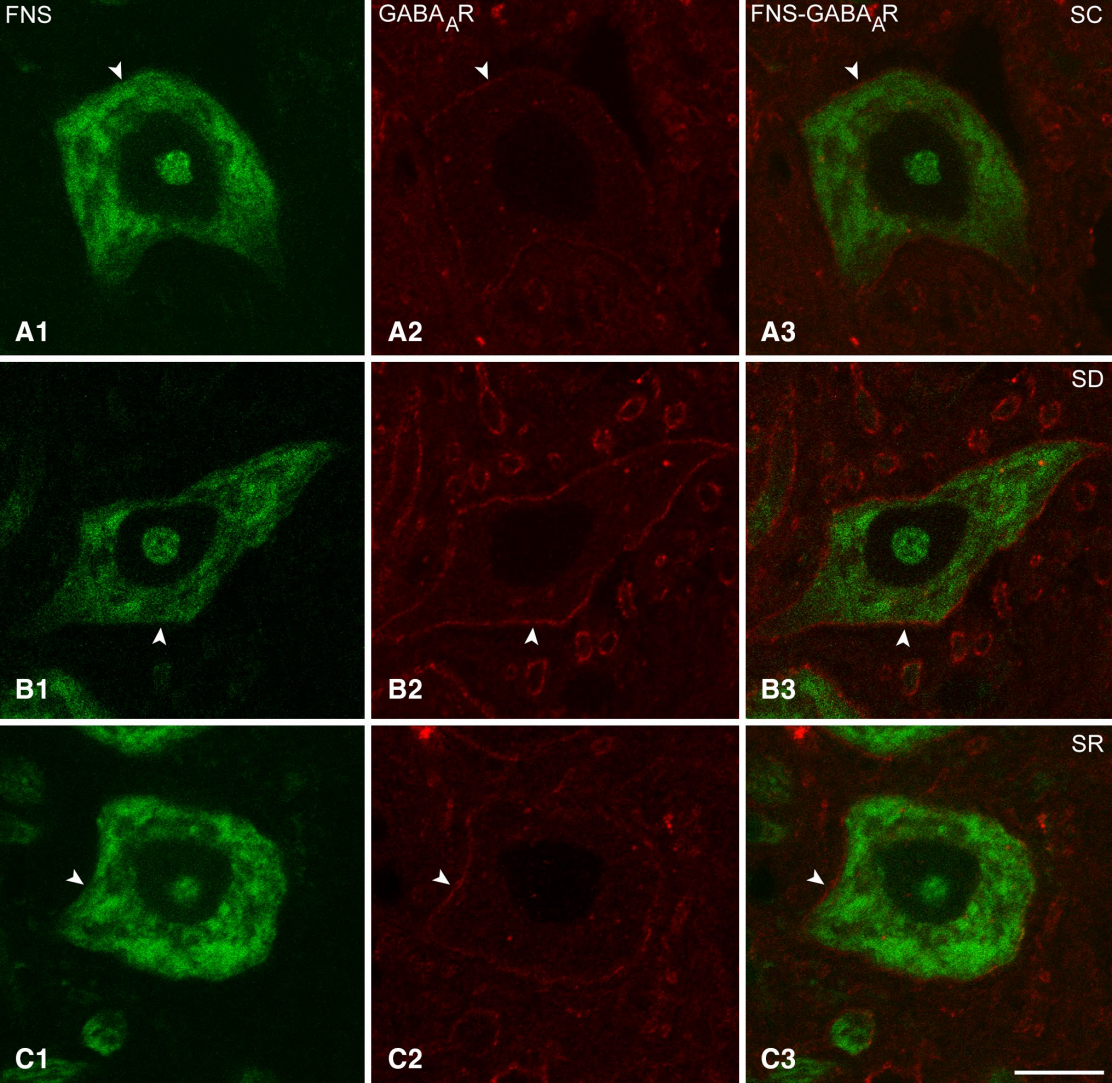
Confocal microscopic images of GABA_A_Rs in Mo5 neurons across groups. Images of single optical sections show large Mo5 neurons stained for Nissl with FNS (*green*, **A1, B1, C1**) and immunostained for the GABA_A_R (*red*, indicated by *arrowheads*) in single (**A2, B2, C2**) and merged images (**A3, B3, C3**). The GABA_A_R immunofluorescence is evident over the plasma membrane, as very dim in the SC mouse (MST24), as very bright in the SD mouse (MST2), and as less bright in the SR mouse (MST11) than in the SD mouse. In all cases, the immunostaining is relatively continuous though with nonuniform intensity along the plasma membrane of the soma and proximal dendrites of the Mo5 neurons. Note that particularly in the SD mouse, the GABA_A_R immunostaining is prominent on the membrane of large dendrites cut in cross section. *Scale bar* 20 μm. Optical image thickness: 0.5 μm

Immunostaining for the GABA_A_R was examined on the Mo5 neurons, which were positively (+) stained for Nissl substance with FNS and morphologically identified as motor neurons (MoFNS+) in brains of mice from SC, SD and SR groups (Fig. 2A–C). As evident in the fluorescent microscopic images examined for determining GABA_A_R positive (+) immunostaining and measuring its intensity (Fig. 2A–C) and in confocal microscopic images examined for its localization with higher resolution (Fig. 3A–C), the GABA_A_R immunostaining was located over the plasma membrane of the soma and dendrites of the MoFNS + neurons. As evident in these images, the immunofluorescent staining varied in intensity among neurons and mice yet appeared to be consistently most intense in neurons of the SD mice.

As determined in the fluorescent microscopic image stacks of optical sections (Fig. 2A–C), a proportion of the MoFNS + neurons appeared to be positively immunostained for the GABA_A_R over the plasma membrane in all mice (Fig. 1B1). The proportion of GABA_A_R+/MoFNS + neurons, however, differed significantly between groups (F_(2,9)_ = 6.1, *p* = 0.021; *n* = 3–6 mice per group) and was significantly greater in the SD group (65.34 ± 6.57%) as compared to the SC group (32.34 ± 7.38%, *post hoc* paired comparison *p* = 0.008) and in a trend as compared to the SR group (47.38 ± 3.27%, *p* = 0.096). As measured in the same fluorescent microscopic image stacks (Fig. 2A–C), the average luminance measures of the GABA_A_R immunostaining on the plasma membrane of GABA_A_R+/MoFNS + neurons was also significantly different between groups (F_(2,110)_ = 10.9, *p* < 0.001; *n* = 23–60 cells per condition, Fig. 1B2) and was significantly higher in SD (45.63 ± 2.61) as compared to SC (36.26 ± 2.53, *post hoc* paired comparison, *p* = 0.023) and SR groups (28.67 ± 1.84, *p* < 0.001).

### GABA_B_Rs on Mo5 neurons after SD and SR

**Fig. 4:**
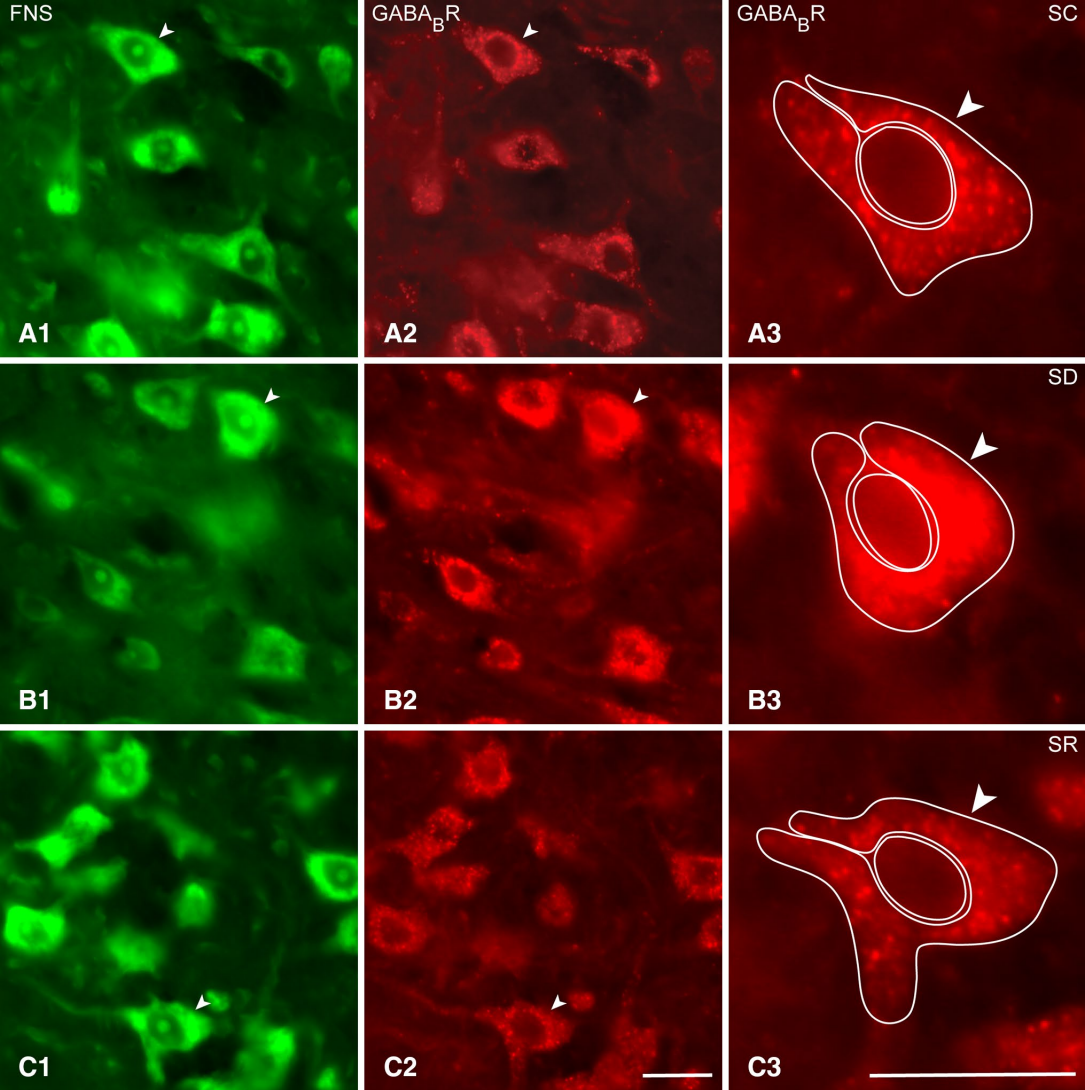
Fluorescent microscopic images of GABA_B_Rs in Mo5 neurons across groups. Images of single optical sections show several large Mo5 neurons stained for Nissl with FNS (*green*, **A1, B1, C1**) and immunostained for the GABA_B_R (*red*, **A2, B2, C2**) within the Mo5 nucleus of each mouse. Through the one optical section shown, GABA_B_R immunofluorescence is clearly visible over the cytoplasm of all Mo5 neurons of which one representative one is magnified for each mouse (**A3, B3, C3**, indicated by *arrowheads*). In the SC mouse (MST21), the GABA_B_R immunofluorescence is moderately bright, whereas in the SD mouse (MST20), it is very bright, and in the SR mouse (MST8), it is also moderately bright and similar to that in the SC mouse. For measurement of the intensity of the GABA_B_R immunofluorescence, a donut-shaped contour was traced around the perikaryon to include all the cytoplasm and plasma membrane, and for that of the background fluorescence for subtraction, a spherical contour to contain the nucleus of each cell (**A3, B3, C3**, see Methods). *Scale bars* 20 μm. Optical image thickness: 0.5 μm

**Fig. 5:**
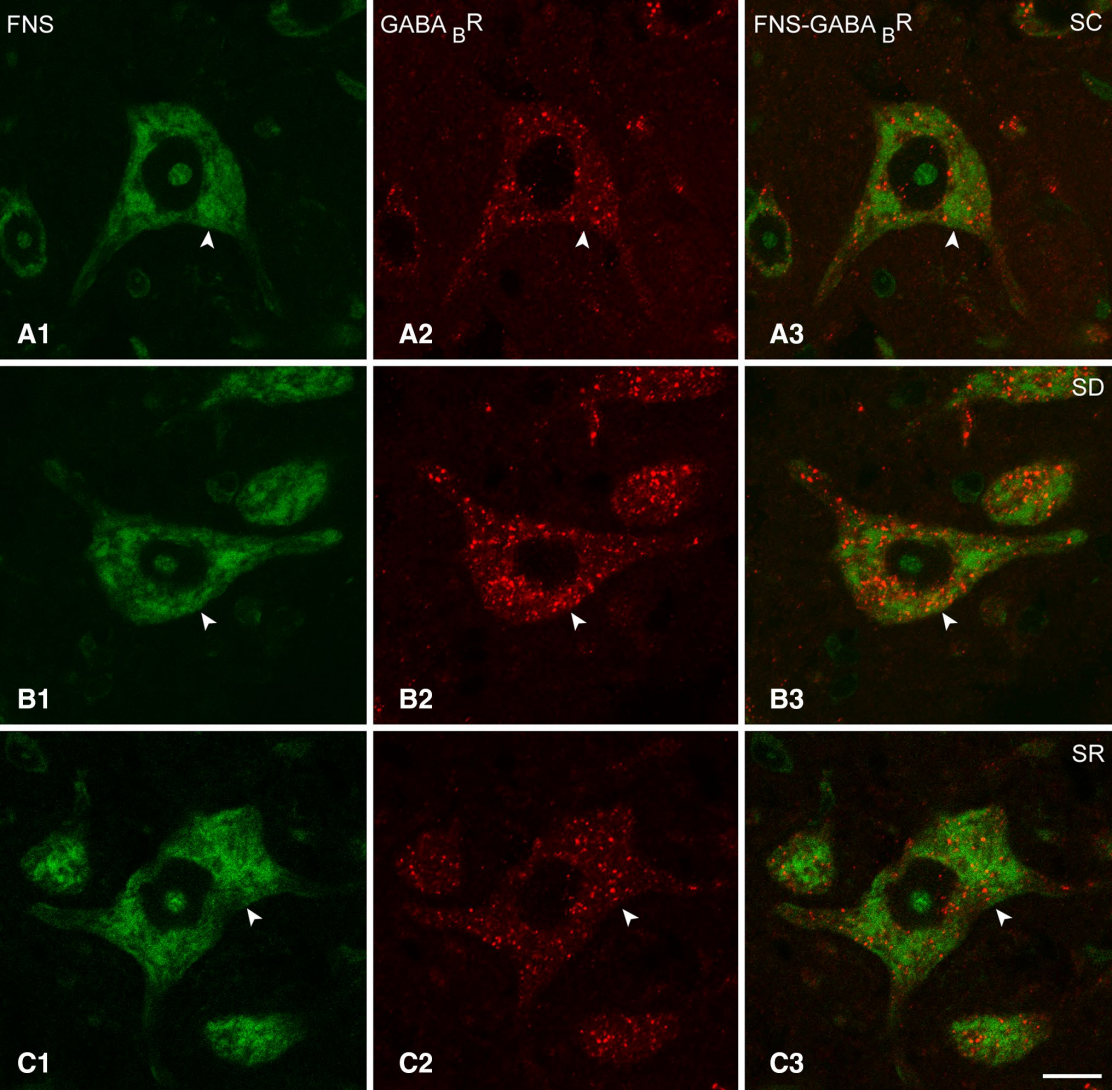
Confocal microscopic images of GABA_B_Rs in Mo5 neurons across groups. Confocal images of fluorescent stained sections show Mo5 neurons stained for Nissl with FNS (*green*, **A1, B1, C1**) and immunostained for the GABA_B_R (*red*, indicated by *arrowheads*) in single (**A2, B2, C2**) and merged images (**A3, B3, C3**). In the mice from all three groups, the GABA_B_R immunofluorescence is visible as small to large granules or vesicles, which are distributed through the cytoplasm out to the plasma membrane of the Mo5 neurons. Differing in density and intensity, these granules appear to be more dense and bright in the SD mouse (MST20), as compared to the SC mouse (MST21). In the SR mouse (MST11), they are lesser than in the SD mouse. *Scale bars* 20 μm. Optical image thickness: 0.5 μm

Immunostaining for the GABA_B_R was examined on the Mo5FNS + neurons in brains from mice of the SC, SD and SR groups (Fig. 4A–C). As evident in the fluorescent microscopic images and in the higher resolution confocal microscopic images of the same material (Fig. 5A–C), GABA_B_R immunostaining appeared over granules or vesicles of different sizes which were evident through the cytoplasm extending from the nucleus out to the periphery and in some cases near the plasma membrane of the soma and proximal dendrites. The specific association of these granules with the plasma membrane, however, could not be adequately resolved to be distinguished from the granules distributed through the cytoplasm. The density and intensity of the granular immunostaining through the cytoplasm however appeared to vary across neurons and mice and to be consistently greatest in the SD group of mice.

As determined in the fluorescent microscopic image stacks of optical sections (Fig. 4A–C), all MoFNS + neurons were judged to be positively immunostained for the GABA_B_R through their cytoplasm in all groups (100%, *n* = 3–6 mice per group, Fig. 1C1). On the other hand, as measured in the same fluorescent microscopic image stacks (Fig. 4A–C), the luminance of the GABA_B_R immunofluorescence in the perikarya of the GABA_B_R+/MoFNS + somata was significantly different between groups (F_(2,117)_ = 9.35, *p* < 0.001; *n* = 30 to 60 cells per group) (Fig. 1C2) being significantly higher in the SD (46.97 ± 2.11) as compared to the SC (32.26 ± 2.99, *post hoc* paired comparison *p* < 0.001) and SR groups (36.45 ± 3.07, *p* = 0.005).

### AChM2Rs on Mo5 neurons after SD and SR

**Fig. 6:**
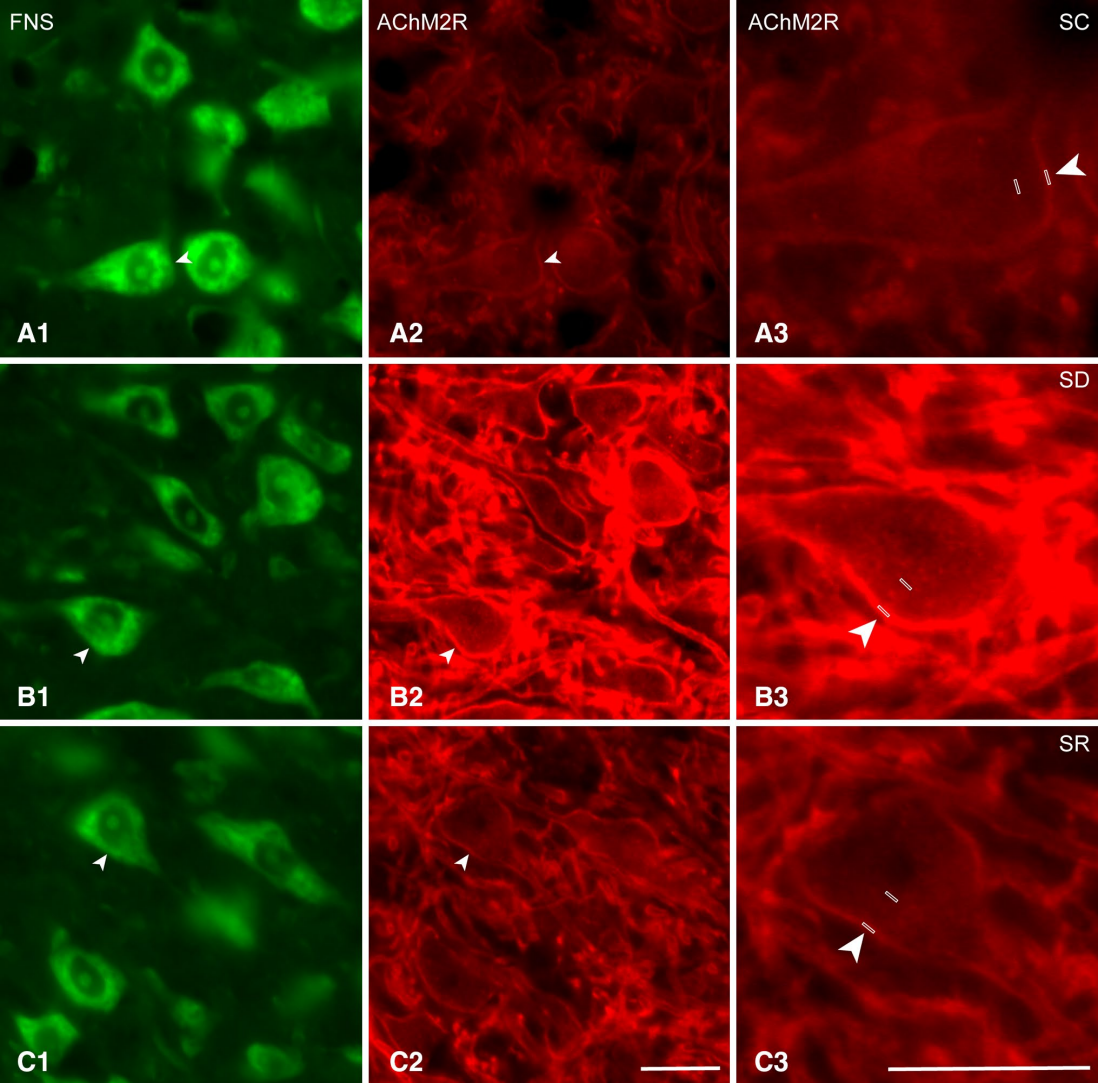
Fluorescent microscopic images of AChM2Rs in Mo5 neurons across groups. Images of single optical sections show several motor neurons stained for Nissl with FNS (*green*, **A1, B1, C1**) and immunostained for the AChM2R (*red*, **A2, B2, C2**) within the Mo5 nucleus of each mouse. Through one optical section, AChM2R immunofluorescence is noticeably visible on the membrane of all Mo5 neurons, of which one is magnified for each mouse (**A3, B3, C3**, indicated by filled *arrowheads*). In the SC mouse (MST21), the AChM2R immunofluorescence is relatively dim, whereas in the SD mouse (MST2), it is very bright, and in the SR mouse (MST22), it is less bright than in the SD mouse. For measurement of the intensity of the AChM2Rs immunofluorescence, a small *rectangular box* was placed over the plasma membrane and for that of the fluorescence background for subtraction, another over the nucleus of each cell (**A3, B3, C3**, see Methods). *Scale bars* 20 μm. Optical image thickness: 0.5 μm

**Fig. 7:**
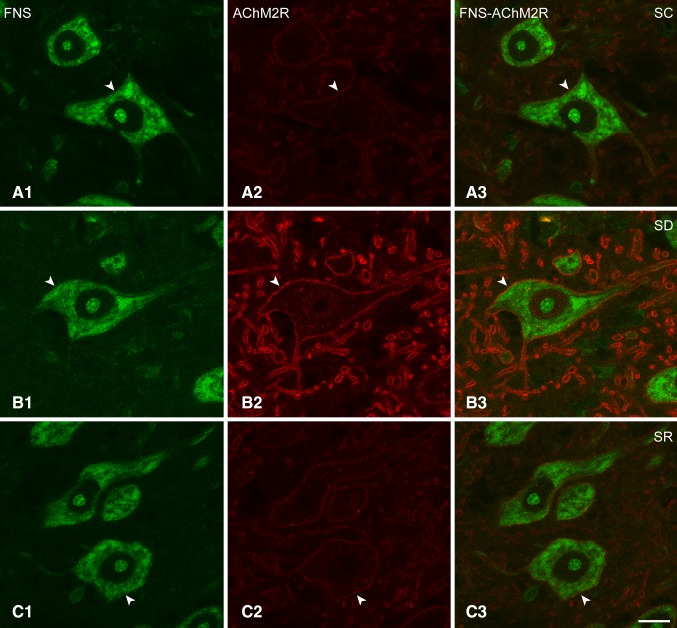
Confocal microscopic images of AChM2Rs in Mo5 neurons across groups. Images of single optical sections show large Mo5 neurons stained for Nissl with FNS (*green*, **A1, B1, C1**) and immunostained for the AChM2R (*red*, indicated by *arrowheads*) in single (**A2, B2, C2**) and merged images (**A3, B3, C3**) for each mouse. The AChM2R immunofluorescence is evident over the plasma membrane in all three mice, though just barely visible in the SC mouse (MST21). In the SD mouse (MST2), the AChM2R staining is very bright and clearly visible along the full membrane of the soma and proximal dendrites. In the SD mouse, it is also prominent on the plasma membrane of the large dendrites which are cut in cross section. In the SR mouse (MST8), the staining is less bright than in the SD mouse. *Scale bars* 20 μm. Optical image thickness: 0.5 μm

Immunostaining for the AChM2R was examined on the MoFNS + neurons across the three groups of mice (Fig. 6A–C). As evident in the fluorescent microscopic images and in the higher resolution confocal microscopic images of the same material (Fig. 7A–C), the AChM2R immunostaining was observed over the plasma membrane of the soma and dendrites of the Mo5 neurons. It appeared to extend along the entire membrane of most cells though to vary in intensity, appearing most intense in the brains of the SD group of mice.

As determined in the fluorescent microscopic image stacks of optical sections (Fig. 6A–C), all MoFNS + neurons were judged to be positively immunostained for the AChM2R on the plasma membrane of the soma and proximal dendrites (100%, *n* = 3 to 6 mice per group, Fig. 1D1). On the other hand, as measured in the same fluorescent microscopic image stacks (Fig. 6A–C), the luminance of the AChM2R immunofluorescence over the membrane of the AChM2R+/MoFNS + cells was significantly different between groups (F_(2,117)_ = 16.34, *p* < 0.001; *n* = 30 to 60 cells per group, Fig. 1D2) and was significantly greater in the SD (96.41 ± 3.90) as compared to SC (58.71 ± 4.27, *post hoc* paired comparison *p* < 0.001) and SR groups (75.47 ± 6.56, *p* = 0.002).

## Discussion

The present results indicate that GABA_A_, GABA_B_ and AChM2 receptors increase in Mo5 neurons with enforced waking during the day when mice normally sleep the majority of the time. The results suggest that the Mo5 neurons undergo homeostatic down-scaling in their excitability through increases in inhibitory receptors following prolonged activity during SD.

### Homeostatic regulation through GABA_A_Rs

In immunofluorescent stained sections, GABA_A_Rs were apparent on the plasma membrane in a proportion of the Mo5 neurons, which was much higher in the SD group than in the SC group, and for which the luminance or density was also higher, indicating increases in membrane GABA_A_Rs following enforced waking and presumed prolonged activity during SD. These increases following SD are similar to those seen on orexin neurons, which are also active during waking and silent during sleep. They are opposite to decreases in GABA_A_Rs seen on MCH neurons, which are silent during waking and active during sleep (Toossi et al. 2016b). The increases in GABA_A_Rs following SD are also similar to the increases in GABA_A_Rs seen *in vitro* and *in vivo* on cortical neurons pharmacologically or electrophysiologically stimulated to fire continuously at high rates (Marty et al. 2004; Nusser et al. 1998). Moreover, increases in the density of the GABA_A_Rs in these experiments were shown to be associated with increases in inhibitory postsynaptic currents (IPSCs). The increased clusters of the GABA_A_R on the membrane seen here thus likely correspond to increases in functional receptors. Reciprocally, pharmacologically induced cessation of firing in vitro led to a decrease of GABA_A_Rs on the membrane associated with a decrease in miniature IPSCs (Marty et al. 2004; Kilman et al. 2002). Similarly here, the density of GABA_A_Rs was reduced to return to SC levels following SR, when the Mo5 neurons would be mostly silent during increased SWS and REMS.

Pharmacological evidence indicates that the GABA_A_R together with the glycine receptor on motor neurons is implicated in the muscle hypotonia and atonia that respectively occur during SWS and REMS (Brooks and Peever 2012; Morrison et al. 2003; Soja et al. 1987). Indeed, mutant mice with deficient GABA_A_ and glycine receptors present symptoms of incomplete muscle atonia and motor inhibition with aberrant twitches and movements during SWS and REMS that resemble the symptoms of REMS behavior disorder in humans (Brooks and Peever 2011). Hypertonic mutant mice were also shown to harbor deficient GABA_A_Rs in the CNS and on motor neurons, such as to suggest that dysregulation of GABA_A_R homeostasis can cause hypertonia in multiple clinical disorders (Gilbert et al. 2006). Here, we present evidence that GABA_A_Rs are normally homeostatically regulated on motor neurons according to activity and state.

### Homeostatic regulation through GABA_B_Rs

GABA_B_Rs were apparent through the cytoplasm of all Mo5 neurons; however the luminance of the GABA_B_Rs was higher in SD than in the SC and SR groups. Given a lack of resolution to distinguish receptor labeling over the plasma membrane, the increased immunostaining for the R1 subunit cannot be inferred to represent an increase in functional GABA_B_Rs but only an increased expression or trafficking of these receptors within intracellular organelles. Nonetheless, the increased expression during SD suggests a contribution of the GABA_B_R to homeostatic down-scaling in the excitability of the motor neurons with enforced waking. We recently found that SD induced a similar increase of GABA_B_Rs in orexin neurons which fire during waking, whereas it induced a decrease in GABA_B_Rs on MCH neurons, which are silent during waking (Toossi et al. 2016b).

Pharmacological evidence has shown that GABA_B_Rs in addition to the glycine and GABA_A_ receptors are implicated in muscle hypotonia and atonia of sleep and that only with antagonism of all three receptors can complete muscle atonia be prevented (Brooks and Peever 2012). *In vitro* evidence has indicated that the GABA_B_R is essential for homeostatic regulation of firing within hippocampal circuits (Vertkin et al. 2015). Compensatory increases in GABA_B_R immunostaining have been reported in dentate gyrus of mice in response to recurrent seizures (Straessle et al. 2003). Genetic deletion of the GABA_B_R results in a disrupted sleep-waking cycle (Vienne et al. 2010) and hyper locomotor activity along with seizures (Schuler et al. 2001). The present results suggest that homeostatic regulation of the GABA_B_R in motor neurons may be important in down-scaling their excitability during prolonged activity with SD.

### Role of GABA in regulating muscle tone across sleep-wake states

GABA and GABAergic neurons have been shown to play roles in sleep including SWS, PS or REMS and associated muscle hypotonia or atonia (Boissard et al. 2002; Holmes and Jones 1994; Maloney et al. 1999, 2000; Xi et al. 1999; Jones 1991; Weber et al. 2015; Krenzer et al. 2011). Juxtacellular recording and labeling of neurons located in the region of the laterodorsal and sublaterodorsal tegmental nuclei (LDT and SubLDT) identified GABAergic neurons that fire at increasingly higher rates during SWS and PS relative to waking and in negative correlation with neck muscle tone (Boucetta et al. 2014). Motor neurons receive GABAergic and glycinergic input from local interneurons in the brainstem and spinal cord. They also receive such input from projecting neurons as was demonstrated in the Mo5 neurons as monosynaptic inhibitory input from neurons in the medullary reticular formation (Chase et al. 1984), where both GABAergic and glycinergic neurons are located (Holmes et al. 1994; Jones et al. 1991; Rampon et al. 1996). They discharged at progressively higher rates during SWS and REM as compared to waking in a manner reciprocally related to the increasing inhibition of the Mo5 neurons during sleep (Chase et al. 1984) and thus in a manner similar to identified GABAergic neurons in the pons (Boucetta et al. 2014). Such identified GABAergic pontine and presumed GABAergic (or glycinergic) medullary neurons could thus be responsible for the progressive inhibition of motor neurons during sleep, culminating in that during PS or REMS.

Here, the increase in both GABA_A_ and GABA_B_ receptors following prolonged waking would suggest that motor neurons would become progressively more responsive to GABA released during waking and sleep and accordingly more susceptible to inhibition and resulting muscle hypotonia and atonia with SD. Such homeostatic changes could underlie the increases in muscle atonia that occur during SWS following SD (Werth et al. 2002) and the increased propensity to cataplectic attacks and paralysis with sleep deficiency or disruption in patients having narcolepsy with cataplexy (Nishino and Mignot 1997). Moreover, daytime cataplectic attacks can be prevented in these patients by administration during the night of gamma hydroxybutyrate (GHB), a GABA_B_R agonist, which allows consolidation of sleep during the night (Boscolo-Berto et al. 2012), presumably associated as suggested here, with decreases of GABA_B_Rs to normal levels on motor neurons.

### Homeostatic regulation through AChM2Rs

Although AChM2Rs were apparent on the plasma membrane of all Mo5 neurons, the luminance of the AChM2Rs markedly increased following SD, presumably due to prolonged activity of the Mo5 neurons during enforced waking, and then returned to SC levels following SR, presumably due to quiescence of the Mo5 neurons during sleep. These changes are interpreted as a homeostatic response to enhanced neuronal activity, which to our knowledge, have not previously been described for AChMRs.

Pharmacological evidence has indicated that ACh participates in the muscle atonia of REMS through M2 receptors on Mo12 neurons (Grace et al. 2013a). Acting upon GIRK channels, ACh inhibits spinal motor neurons through M2Rs (Chevallier et al. 2006; Miles et al. 2007), which have also been visualized here and in previous studies upon the postsynaptic membrane of Mo5 neurons opposite cholinergic terminals (Hellstrom et al. 2003; Brischoux et al. 2008). The Mo5 neurons could thus be homeostatically regulated following enforced waking by down-scaling through increases in inhibitory AChM2Rs. Such increases in these receptors would render the motor neurons more susceptible to inhibition by ACh and thus the muscles to atonia.

### Role of ACh in regulating muscle tone across sleep-wake states

ACh has long been known to play an important role in waking and PS or REMS, including muscle atonia as evidenced by severe reduction or elimination of PS following lesions of the LDT/PPT cholinergic neurons (Webster and Jones 1988) and induction of PS or REMS by administration of cholinergic agonists into the pontine reticular formation, an effect which is dependent upon AChM2Rs (Baghdoyan and Lydic 1999; Velazquez-Moctezuma et al. 1989). ACh can apparently play this role in part through direct influence on motor neurons, which for the Mo5 and Mo12, have been shown to receive input from cholinergic neurons of the LDT and pedunculopontine tegmental (PPT) nuclei and of the medullary reticular formation (Jones 1990; Woolf and Butcher 1989; Rukhadze and Kubin 2007; Fort et al. 1990). Cholinergic neurons of LDT/PPT discharge during waking and PS and are silent during SWS (Boucetta et al. 2014). They could thus exert an inhibitory influence through AChM2Rs during PS, but also during waking. According to the pharmacological studies on Mo12 neurons, however, antagonism of muscarinic receptors only affects the muscle atonia of REMS and not the level of muscle tone during waking or SWS (Grace et al. 2013a), suggesting differences that might in part be due to the density of the AChM2Rs. Accordingly, the homeostatic increase in AChM2Rs on motor neurons following SD could contribute to the increased propensity in waking to cataplexy and paralysis following sleep deficits in patients having narcolepsy with cataplexy (Nishino and Mignot 1997). Indeed, the sleep cycle of these patients is greatly disrupted and often associated with insomnia during the night, which according to our results could result in increased AChM2, along with GABA_A_ and GABA_B_, receptors on motor neurons during the day.

We conclude that Mo5 neurons are homeostatically regulated across the sleep-waking cycle through changes in the inhibitory receptors for GABA and ACh. Their prolonged activity during enforced waking with SD results in homeostatic down-scaling through increases in GABA_A_, GABA_B_ and AChM2Rs and their subsequent quiescence with SR in restorative up-scaling to return the neurons to normal stable levels of excitability and activity. The increases in these inhibitory receptors following SD would render the motor neurons more responsive and thus susceptible to inhibition by GABA and ACh, which would also render the muscles more susceptible to hypotonia or atonia, as can occur with sleep deficits and particularly in association with sleep disorders, such as narcolepsy with cataplexy.
